# A Review of Materia Medica, for the Use of Students

**Published:** 1852-02

**Authors:** 


*A Review of Materia Medica, for the use of Students.* By JOHN B. BIDDLE, M. D., formerly Professor of Materia Medica in the Franklin Medical College of Philadelphia, &c. &c. pp. 322. *With Illustrations.* Philadelphia: Lindsay & Blakiston: 1852.

This little volume is intended as a manual of Materia Medica for the use of Students. There is, perhaps, no branch of medicine in which the absence of elementary text-books, of convenient size, adapted to the subject as taught in our medical schools, has been more felt than in this; and the author has been induced to hope "that a condensed review of the elements of Materia Medica may be found useful, as a guide to the courses of lectures delivered on this department in the United States." The work "makes no pretensions to rank among original treatises, but is designed to present the leading facts and principles usually comprised under this head, as set forth by the standard authorities."—(*Preface, page* 3.)

